# Supplementary material for: Discovery adductomics provides a comprehensive portrait of tissue-, age- and sex-specific DNA modifications in rodents and humans
Source: Nucleic Acids Res. 2023 Oct 16;51(20):10829–45. doi: 10.1093/nar/gkad822 (PMC10639045; doi:10.1093/nar/gkad822)
Supplement: gkad822_Supplemental_Files [file gkad822_supplemental_files.zip › Guilbaud et al Revised Supplementary Information 2023 8-25.pdf]

Supplementary Information

A comprehensive portrait of tissue-, age- and sex-specific DNA modifications in rodents and humans

Axel Guilbaud, Farzan Ghanegolmohammadi, Yijun Wang, Jiapeng Leng, Alexander Kreymerman, Jacqueline Gamboa Varela, Jessica Garbern, Hannah Elwell, Fang Cao, Elisabeth M. Ricci-Blair, Cui Liang, Seetharamsing Balamkundu, Charles Vidoudez, Michael S. DeMott, Kenneth Bedi, Kenneth B. Margulies, David A. Bennett, Abraham A. Palmer, Amanda Barkley-Levenson, Richard T. Lee, Peter C. Dedon

Contents

• **Supplementary Figure S1**: Examples of detection of putative DNA adducts in the discovery phase of stepped MRM.

• **Supplementary Figure S2.** Parameters and data for isotope-dilution chromatography-coupled triple quadrupole mass spectrometric quantification of DNA adducts in rat and human tissues.

• **Supplementary Figure S3.** Multivariate statistical analysis of 52 (**a-c**) and 64 (**d-f**) shared DNA adducts in group #1 and group #2, respectively, rat tissues reveals 5 co-varying clusters based on age, sex, and tissue.

• **Supplementary Figure S4.** High–resolution MS^3 Orbitrap analysis reveals the structure of the putative DNA adduct at *m/z* 455.

• **Supplementary Figure S5.** DNA damage is strongly tissue-specific in rats.

• **Supplementary Figure S6.** Age-biased DNA adducts in four rat tissues in two different sets of rats.

• **Supplementary Figure S7.** Analysis of DNA damage products in rat, mouse, and human tissues.

• **Supplementary Figure S8.** Presence of 1,N^{6}-εA in the nucleus of human cardiomyocytes.

• **Supplementary Table S1.** Characteristics of the human tissue donors.

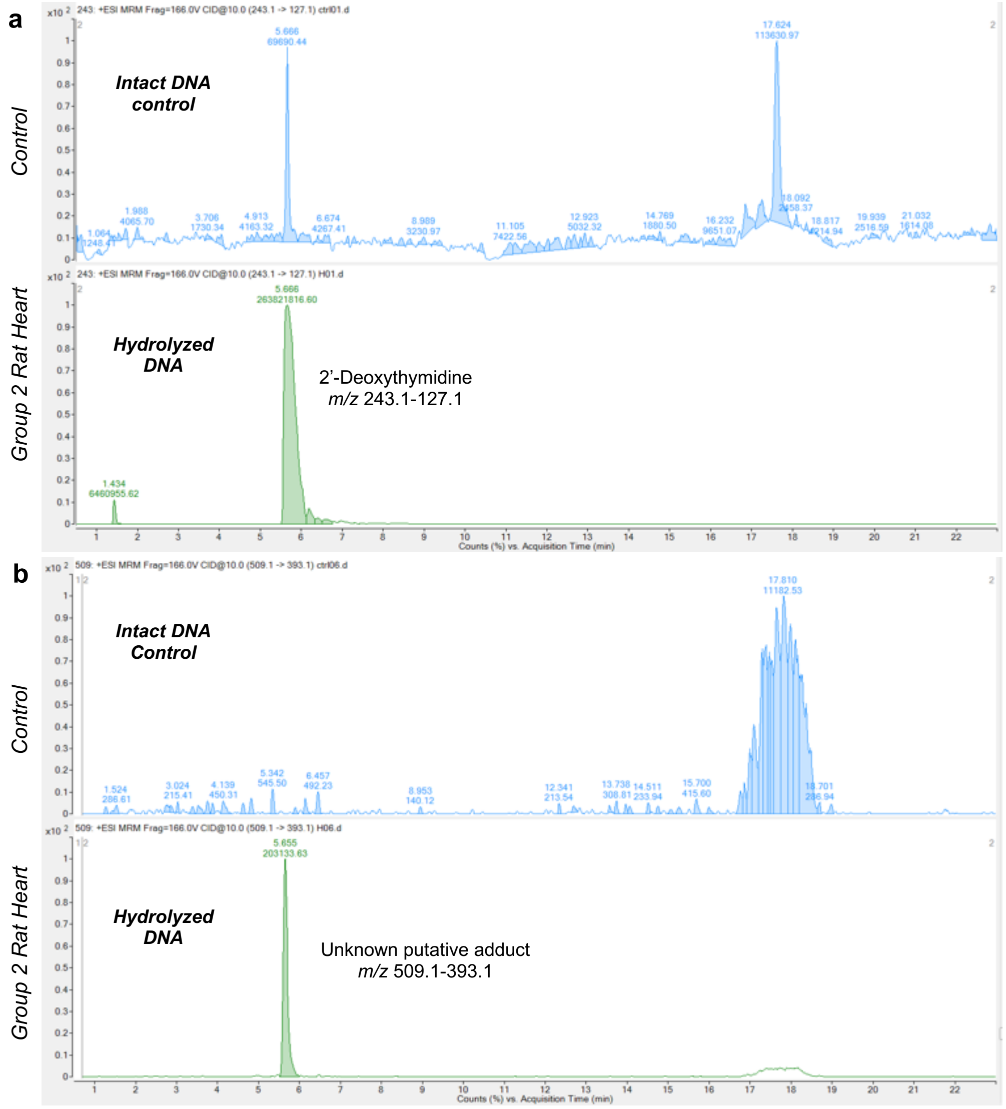

Supplementary Figure S1. Examples of detection of putative DNA adducts in the discovery phase of stepped MRM. Shown are extracted ion chromatograms for (**a**) canonical nucleoside 2’-deoxythymidine (*m/z* 243→127; retention time (RT) = 5.6 min) and (**b**) an undefined adduct (*m/z* 509 →393; RT = 5.6 min; normalized signal intensity = 39.2) in group #2 rat heart. The upper graphs in panels a and b are derived from the intact DNA control injection while the lower panels represent the MRM tracing derived from hydrolyzed DNA containing individual 2’-deoxyribonucleosides. In panel **a**, the presence of apparent 2’-deoxythymidine represents free nucleoside present in the intact DNA sample and represents 0.03% of the 2’-deoxythymidine present in the hydrolyzed DNA sample. Similar results were obtained with the other canonical nucleosides. The late-eluting signals at ~17 min are present in most samples and represent unidentified contaminants eluting significantly later than the latest-eluting putative 2’-deoxyribonucleoside (12 min). Data are derived from raw mass spectrometry data uploaded the the Chorus website (https://chorusproject.org/pages/index.html; accession code 1767) and from **Supplementary Table S2.**

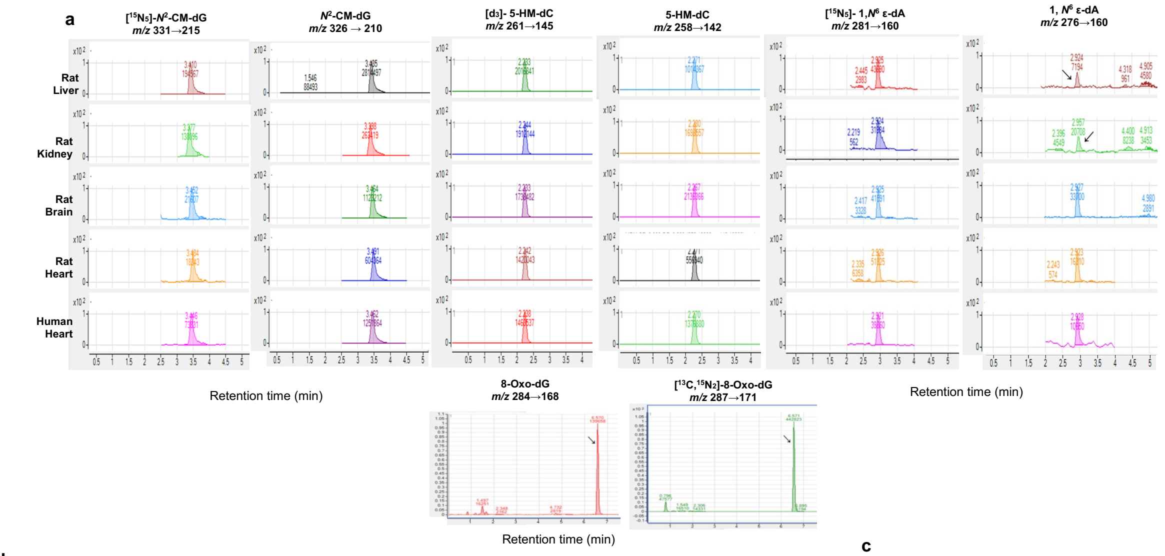

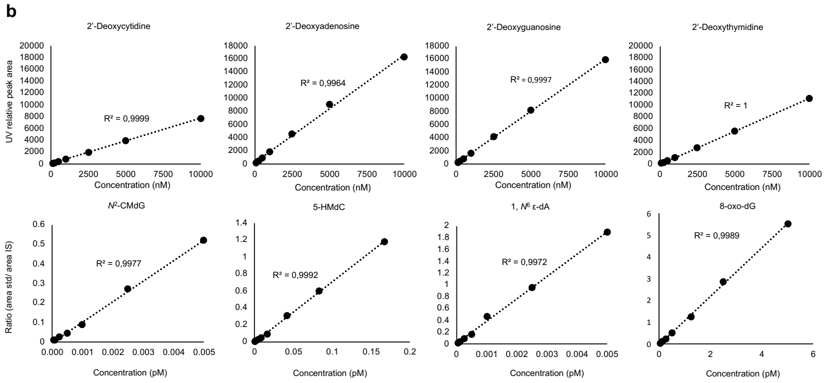

c

	DNA modification name	Abbreviation	Mass, Da	[M + H]+, m/z	[M + H - 116]+, m/z	RT (min)	Corresponds to Adduct?
Deoxycytidines	5-Methyl-2'-deoxycytidine	5-MdC	241	242	126	1.3	YES
	3, N^4-Etheno-2'-deoxycytidine	3, N^4εdC	251	252	136	2.2	YES
	5-Formyl-2'-deoxycytidine	5-FdC	255	256	140	4.6	YES
	5-Chloro-2'-deoxycytidine	5-CldC	261	262	146	4.2	NO
	5-Carboxy-2'-deoxycytidine sodium salt	5-CadC Na^+	293	294	178	2.1	NO
	5-Hydroxymethyl-2'-deoxycytidine	5-HMdC	257	258	142	2.2	YES
Deoxyguanosines	N^2-Carboxymethyl-2'-deoxyguanosine	N^2CMdG	325	326	210	3.4	YES
	Spiroiminodihydantoin	Sp	299	300	184	0.9	NO
	Guanidinohydantoin	Gh	273	274	158	4.2	NO
	2'-Deoxyanthosine	dX	268	269	153	5.7	NO
	Purimidopurinone of 2'-deoxyguanosine	M1dG	303	304	188	8.8	YES
	O^6-Methyl-2'-deoxyguanosine	O^6MdG	281	282	166	9.1	NO
	N^2-Methyl-2'-deoxyguanosine	N^2-MdG	281	282	166	7.3/ 8.1	YES
	8-Oxo-2'-deoxyguanosine	8-oxodG	283	284	168	6.6	YES
	8-Chloro-2'-deoxyguanosine	8-CldG	301	302	186	9.0	NO
Deoxyadenosines	2-Chloro-2'-deoxyadenosine	2-CldA	285	286	170	8.3	NO
	1, N^6-Etheno-2'-deoxyadenosine	1, N^6εdA	275	276	160	2.9	YES
	8-Oxo-2'-deoxyadenosine	8-oxodA	267	268	152	6.9	YES
	8-Chloro-2'-deoxyadenosine	8-CldA	285	286	170	10.4	NO

Supplementary Figure S2. Parameters and data for isotope-dilution chromatography-coupled triple quadrupole mass spectrometric quantification of DNA adducts in rat and human tissues. **(a)** Representative extracted ion chromatograms for analysis of N^2-CMdG, 5-HMdC,8-oxo-dG, and 1,N^6-εdA and their isotope-labeled counterparts in rat and human tissues. **(b)** Illustrative calibration curves for the canonical 2'-deoxyribonucleosides and several DNA adducts. Standard solutions of canonical 2'-deoxyribonucleosides were quantified by UV spectroscopy with an in-line detector and the response factor calculated as the slope of the UV signal versus concentration. For DNA adducts, calibration samples containing fixed amounts of isotope-labeled standard (IS) and variable amounts of unlabeled standard (std) were injected on the LC-MS/MS and the response factor calculated as the slope of the std/IS area ratios versus the std concentration. Ultimately, the adduct quantities are normalized by dividing them by the quantities of canonical 2'-deoxyribonucleosides to correct for sample and injection variability. **(c)** LC-MS/MS parameters for known DNA adducts and modifications analyzed here. These data pertain to **Figures 4** and **5.**

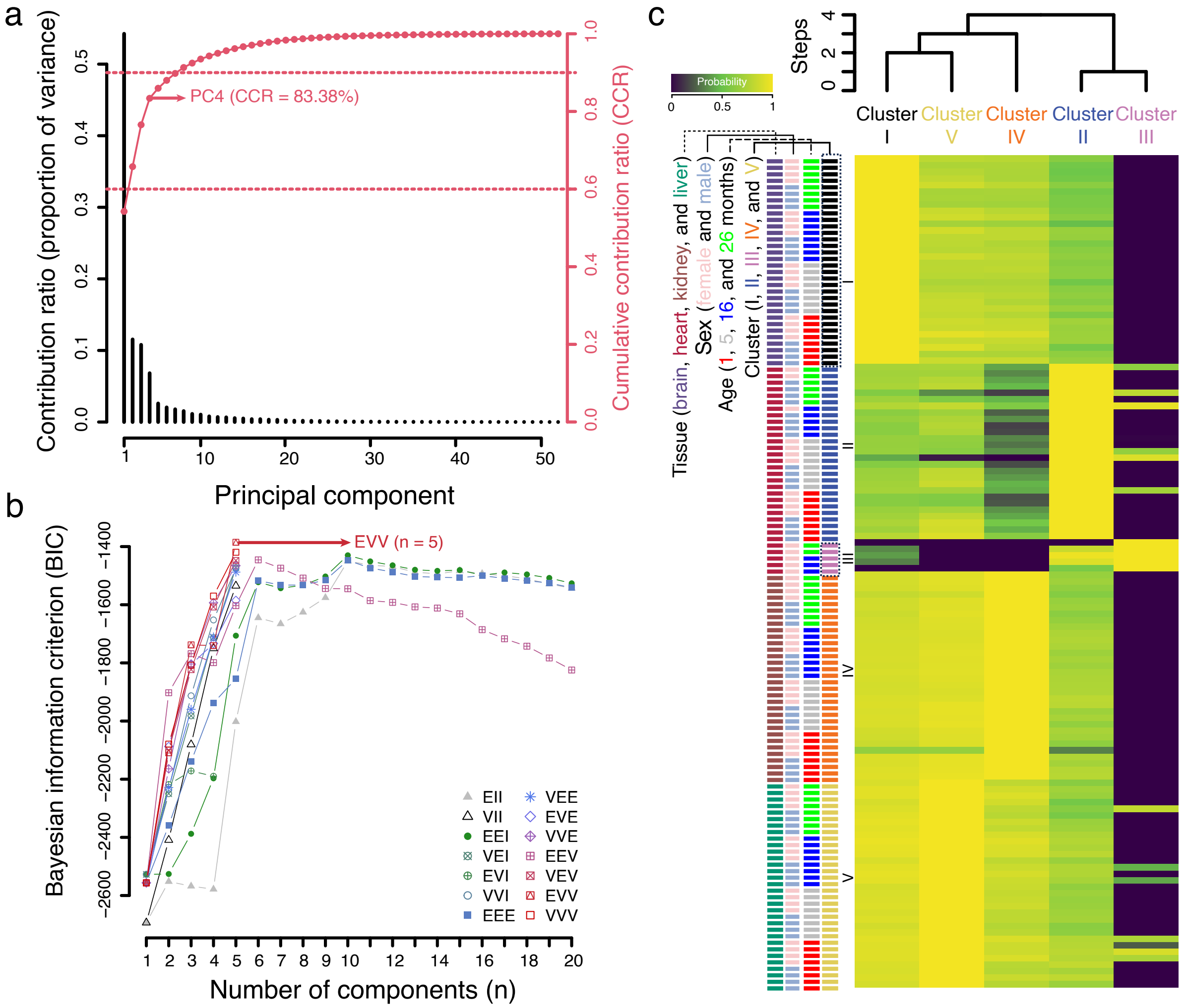

Supplementary Figure S3. Multivariate statistical analysis of 52 **(a-c)** and 64 **(d-f)** shared DNA adducts in group #1 and group #2 rat tissues, respectively, reveals 5 co-varying clusters (I-V) based on age, sex, and tissue. **(a, d)** Principal components analysis. Black bars (left axis) indicating the contribution ratio (proportion of variance), red circles (right axis) indicating the cumulative proportion of variance (cumulative contribution ratio [CCR]), and horizontal dashed lines (right axis) indicating CCRs of 60% and 90%. **(b, e)** Bayesian analysis to define the number of components for Gaussian mixture model (GMM) clustering. The number of components, describing the underlying Gaussian distributions, was defined based on Bayesian information criterion values of models with differing parametrizations. The best fitting model (Ellipsoidal distributions with equal volume and variable shape and orientation axes, EVV) had 5 components that were used for GMM clustering. **(c, f)** Membership probability matrix based on GMM clustering. Each value shows the posterior probability based on the model in panels **b** and **e.** The posterior probability describes the likelihood of each sample belonging to each cluster. Logarithmic transformation of conditional probabilities from expectation maximization was used to generate the heatmap. The dendrogram illustrates model-based hierarchical agglomerative steps based on the Gaussian probability model for maximizing the resulting likelihood. Dashed lines around Cluster I and III adducts on left axis is a visual aid to distinguish colors. This figure is associated with **Figure 3b.**

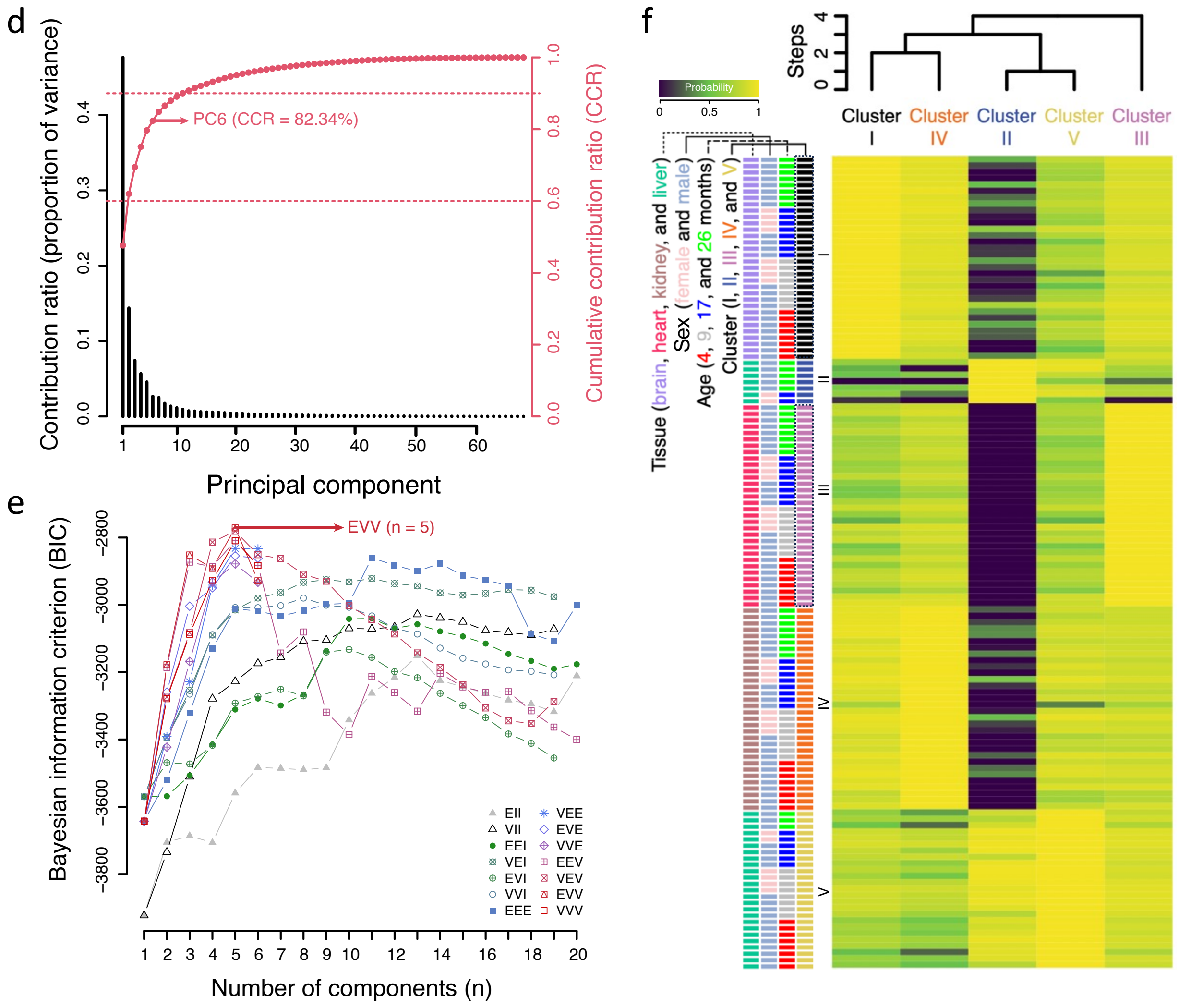

Supplementary Figure S3, continued. See legend above.

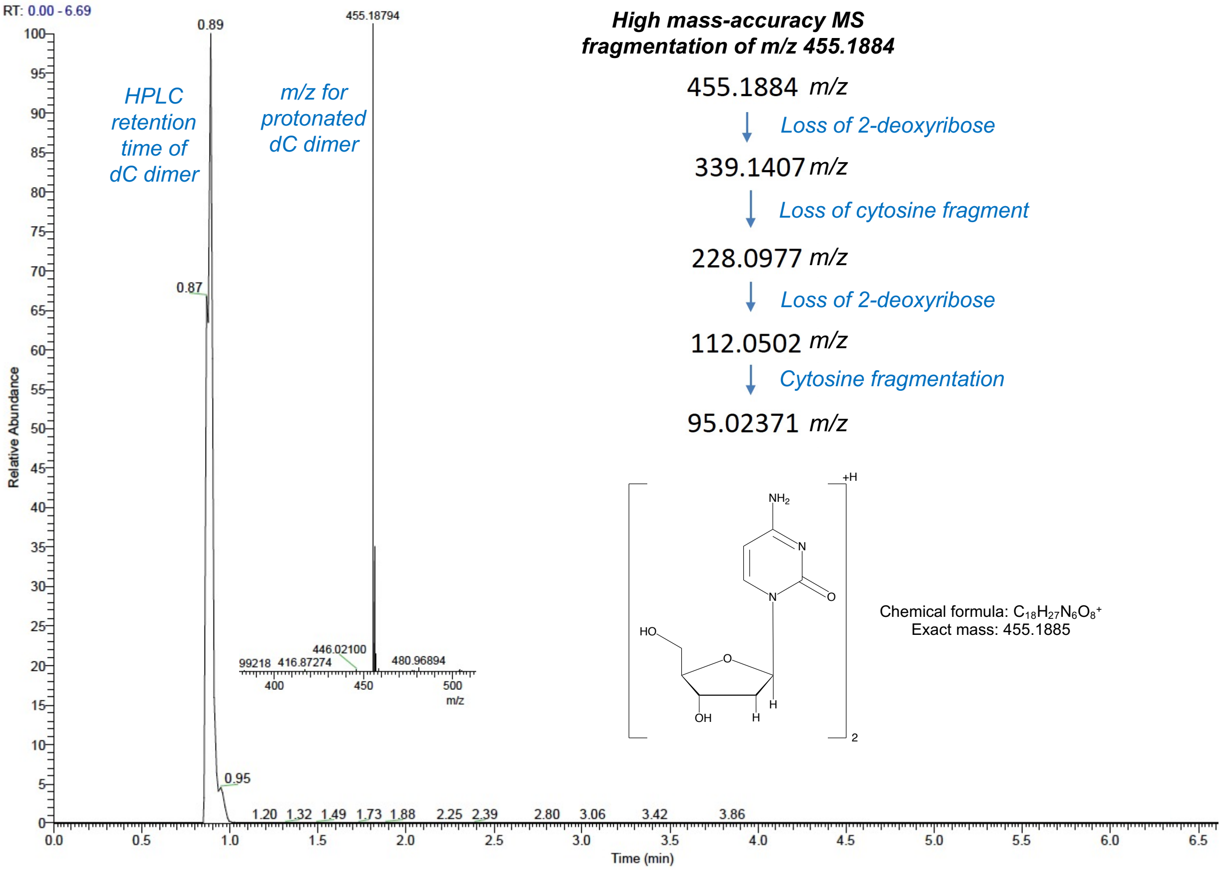

Supplementary Figure S4. High–resolution MS[3] Orbitrap analysis reveals the structure of the putative DNA adduct at *m/z* 455. *Left to right*: The HPLC chromatogram shows retention time of 0.89 min. The mass spectrum shows an *m/z* value of 455.18794 for the protonated species, which equates to an exact mass of 454.1801 for the adduct. The MS[3] fragmentation series shows progressive loss of two cytosine-sized fragments and is consistent with a protonated dC dimer, such as a cyclobutane dimer. These data pertain to **Figure 1** and **Supplementary Table S2**.

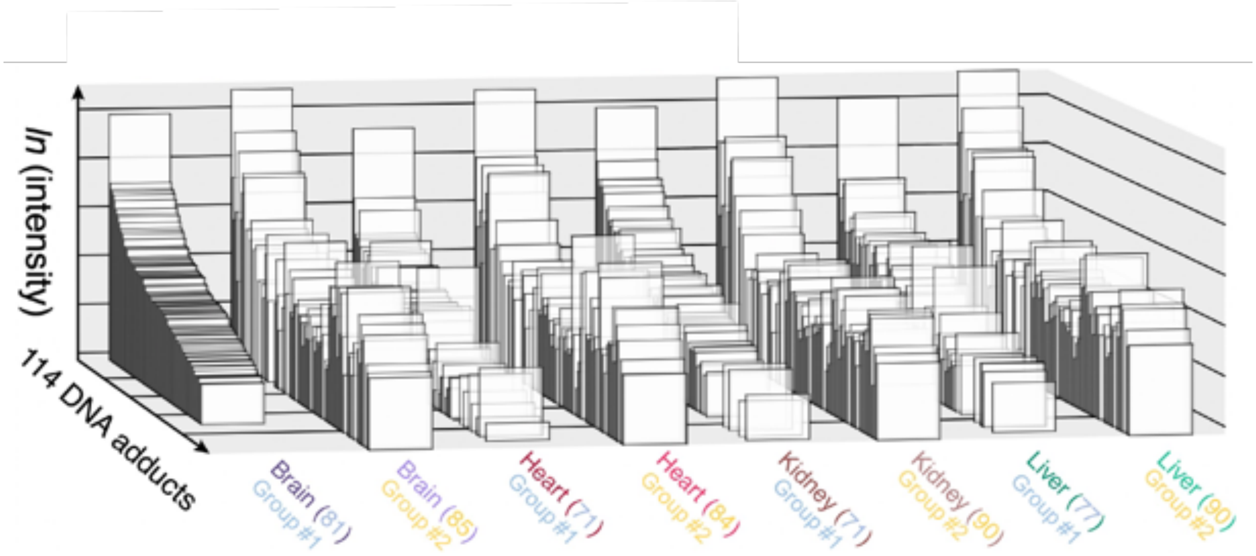

Supplementary Figure S5. Comparison of normalized LC-MS signal intensities for the 114 putative DNA adducts detected in the stepped MRM discovery phase of this study. This set comprises 92 putative DNA adducts in 4 tissues in group #1 rats and 94 in group #2. The ln(signal intensity) rank order of *m/z* values along the z-axis (from **Supplementary Table S2**) was established relative to brain from group #1 rats (left-most plot) with comparisons to heart, kidney, and liver for group #1 and group #2 rats. For each tissue, numbers in parentheses represent the total number of putative DNA adducts detected. These data pertain to **Figures 1** and **3.**

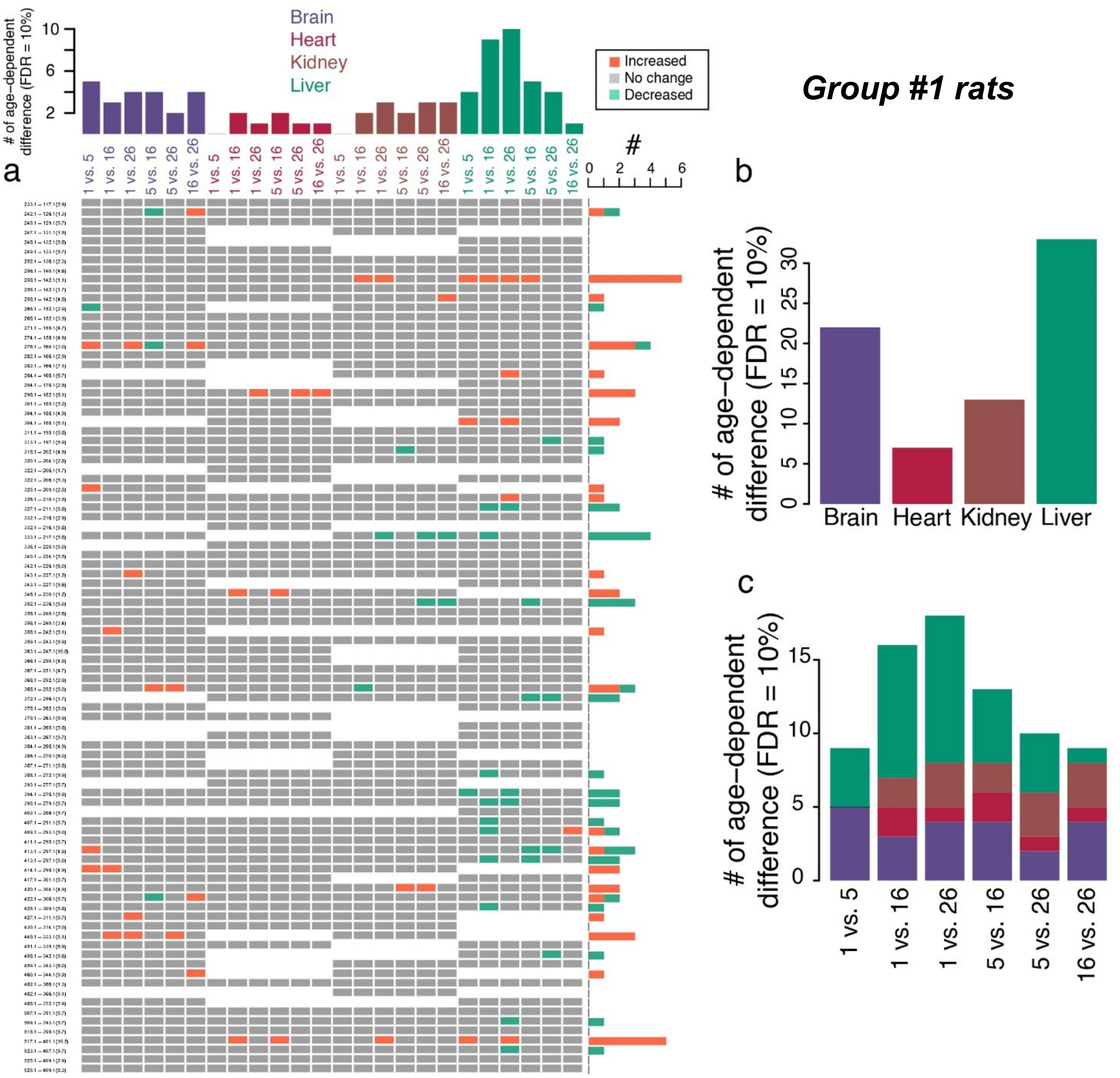

Supplementary Figure S6. Age-biased DNA adducts in four rat tissues in two different sets of rats. Panels **a-c** and **d-f** represent rat groups #1 and #2, respectively. **(a, d)** Heatmaps show pairwise comparisons of all ages in each tissue. Significant changes (FDR = 10%) are presented as red (increase) and green (decrease) boxes. The top bar plot shows the number of significant age-dependent adducts in each age-pairwise comparison; the y-axis is located on the left. The bar plot on the right shows the number of times the adduct is significantly increased (red) or decreased (green) in all tissues. (**b**, **e**) Bar plots of overall frequency of significantly age-biased adducts (FDR = 10%) in each tissue. (**c**, **f**) Bar plots of overall frequency of significantly age-biased adducts (FDR = 10%) in each age-pairwise comparison. These data pertain to **Figures 1** and **4,** and **Supplementary Table S5.**

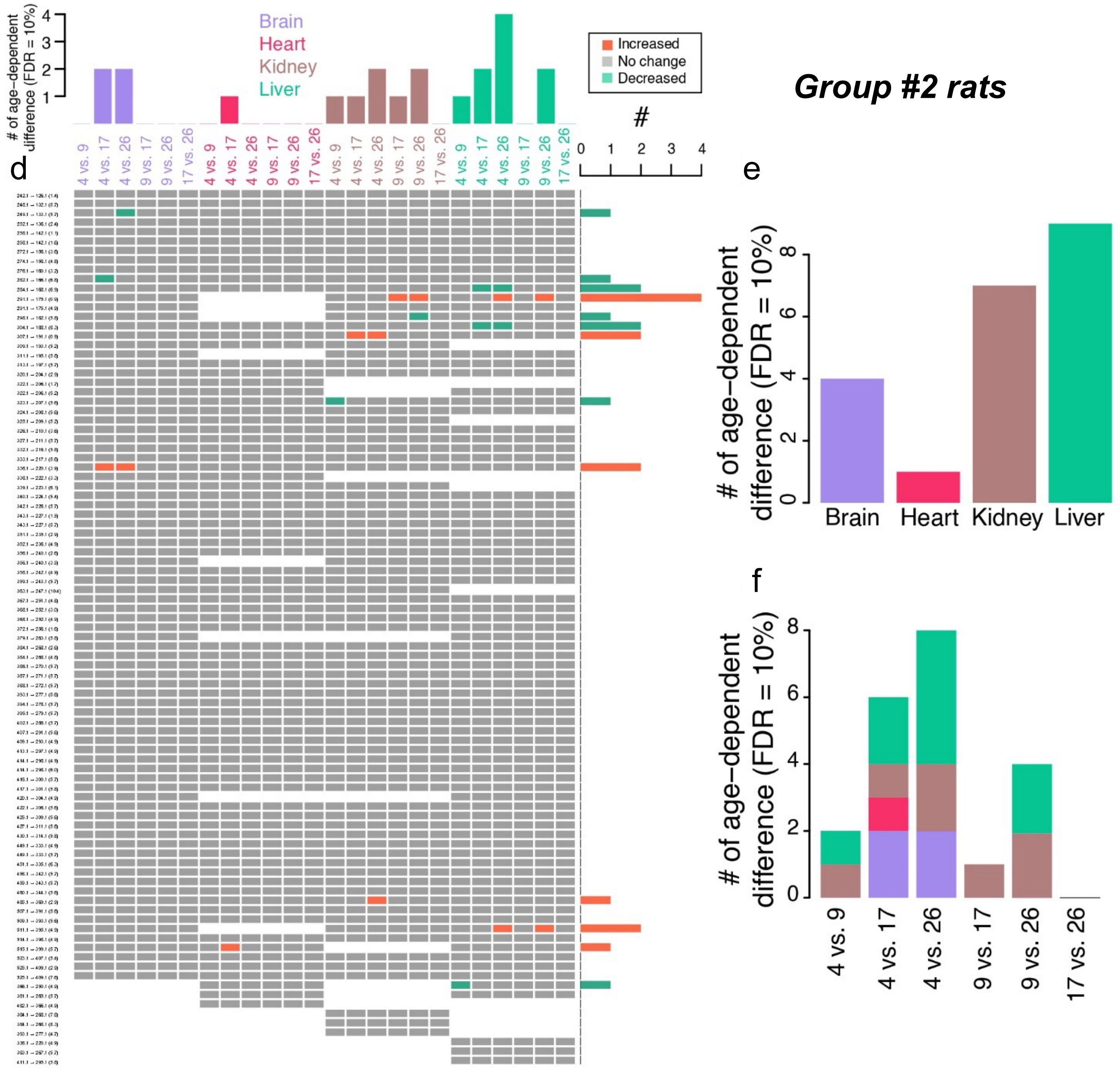

Supplementary Figure S6, continued from previous page. Age-biased DNA adducts in four rat tissues in two different sets of rats.

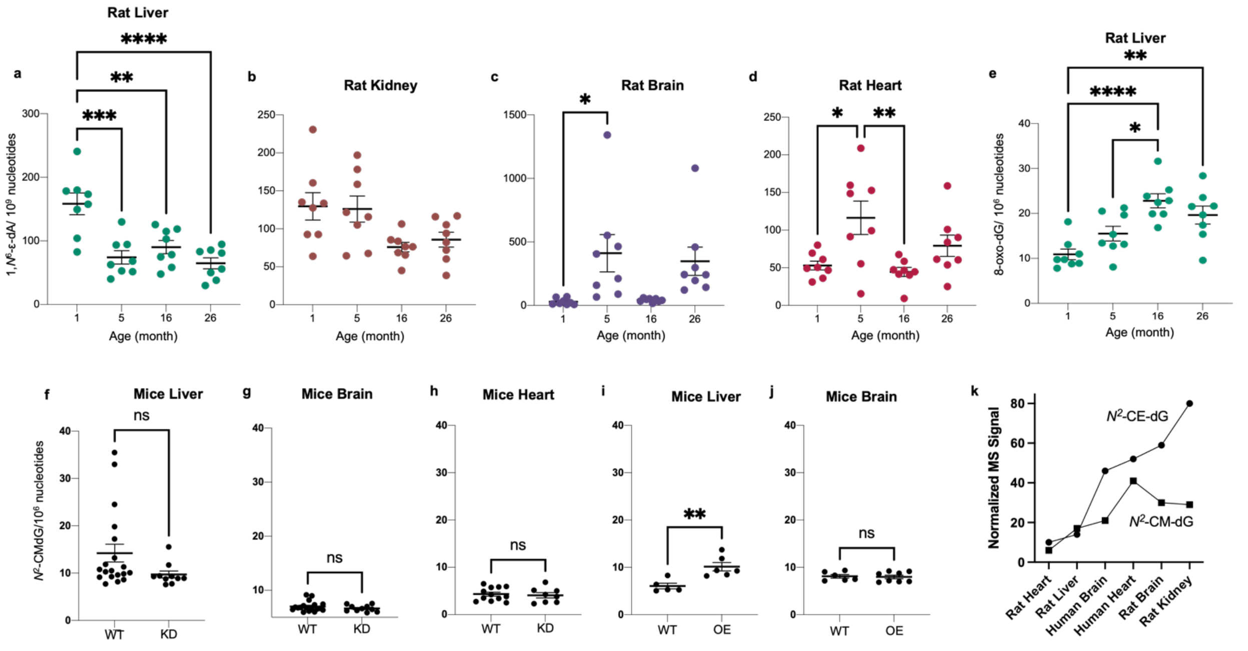

Supplementary Figure S7. Analysis of DNA damage products in rat, mouse, and human tissues. (**a-j**) Isotope dilution chromatography-coupled triple quadrupole mass spectrometric analysis of DNA adducts in rat and mouse tissues. 1,N^6-ε-dA levels in rat tissues (group #1) as a function of age: liver (**a**), kidney (**b**), brain (**c**), and heart (**d**). Data are mean ± SEM for n = 8 rats. One-way ANOVA with Bonferroni's multiple comparisons test was used to evaluate difference among ages., *p<0.05, **p<0.01, ***p<0.001, ****p<0.0001. (**e**) 8-Oxo-dG levels as a function of age in rat liver. Bars represent the average amount of DNA damage with error bars reflecting ± SEM. One-way ANOVA with Bonferroni's multiple comparisons test was used to evaluate differences among ages, *p<0.05, **p<0.01 and **** p<0.0001. Associated with **Figure 3**. (**f-j**) Glyoxalase I levels do not affect the level of N^2-CMdG in transgenic mice. N^2-CMdG levels were measured in liver (**f, i**), brain (**g, j**), and heart (**h**) from glyoxalase I knockdown (KD) mice (**f-h**) and from glyoxalase I over-expressing (OE) mice (**i, j**). (**k**) Correlation between for putative N^2-CE-dG and N^2-CM-dG in rat and human tissues. Normalized signal intensities were taken from **Supplementary Tables S2** (rat) and **S6** (human). (**a-j**) Bars represent the average amount of DNA damage with error bars reflecting ± SEM. A two-tailed unpaired Student's t-test was used to evaluate the difference between wild-type (WT) and transgenic mice (KD, OE). ** p< 0.01, *ns*: not-significant (**f-h**). Plot of normalized MS signal intensities for putative N^2-CE-dG (m/z 340-224) and validated N^2-CM-dG for rat and human tissues. Data represent mean for n=8 measurements of male and female tissues. This figure is associated with **Figure 3**.

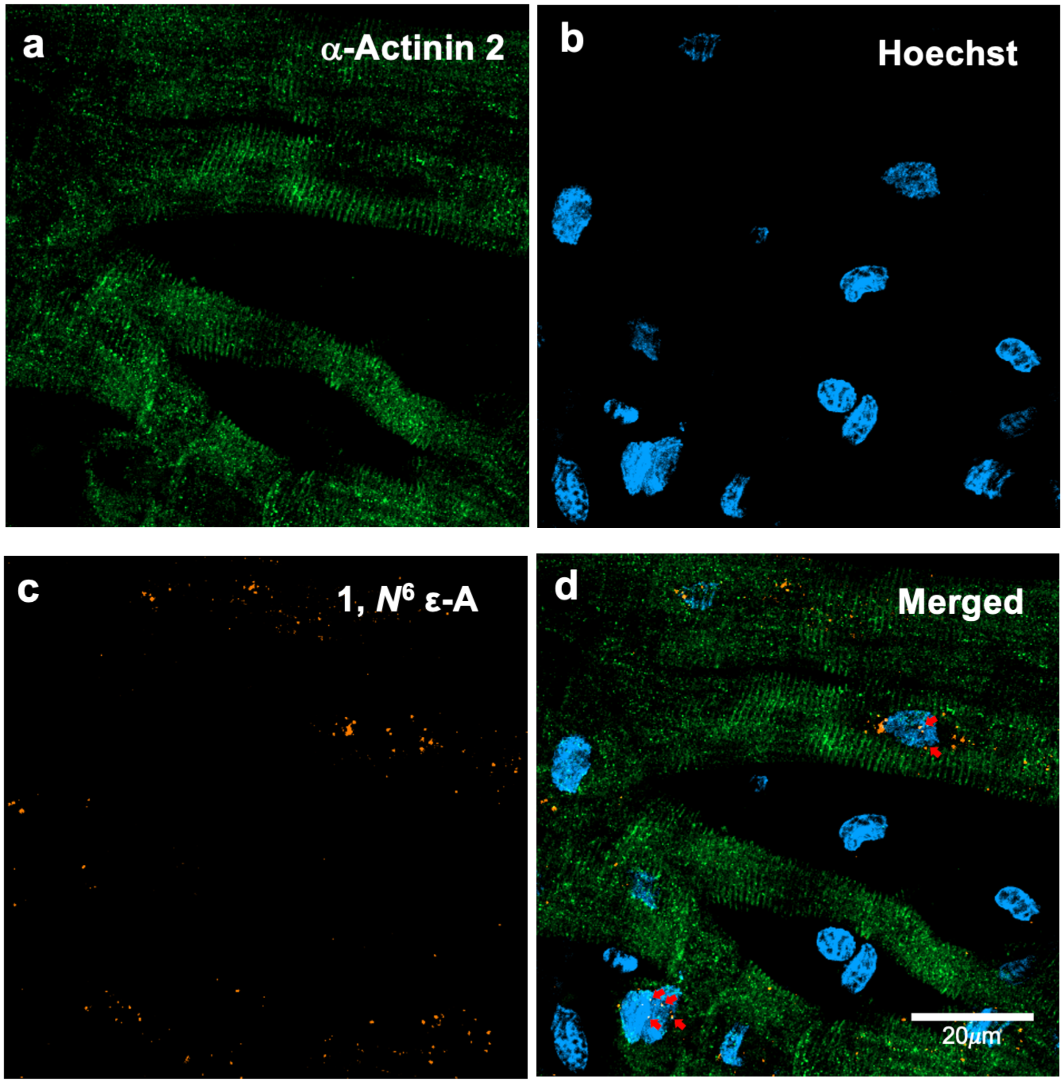

Supplementary Figure S8. Presence of 1,N^{6}-εA in the nucleus of human cardiomyocytes. (**a-d**) Confocal imaging of sectioned and immune-stained human non-failing heart samples reveals the presence of scattered and sparse 1,N^{6}-εA throughout cardiac tissue. 1,N^{6}-εA is present within cardiac cells, as seen by orange spots found within green (α-Actinin 2 labeled) areas. Red arrows denote 1,N^{6}-εA spots within nuclei (labeled with Hoechst), as shown in the merged figure panel (**d**). Scale bar represents a length of 20 μm.

Supplementary Table S1. Characteristics of the human tissue donors. ***Upper: heart tissue.*** LV: Left Ventricle, HMI: Heart Mass Index, LVMI: Left Ventricular Mass Index, BSA: Body Surface Area, BMI: Body Mass Index, LVEDd: Left Ventricle End-Diastolic dimension, LVEDs: Left Ventricle End-Systolic dimension, PW: Posterior Wall, LVEF: Left Ventricle Ejection Fraction. ***Lower: brain tissue.*** Donors are represented according to their ID number (#), category (control, CT), age at death and gender. *n*= 10 CT. These data pertain to **Figure 5**.

Patient ID #	Age (year)	Gender	LV Mass	Heart Weight	% of LV/ Ht weight	HMI	LVMI	Weight (Kg)	Height (cm)	BSA	BMI	LVEDd	LVEDs	PW Thick	LVEF	Creatinine
1584	22	Male	182	299	61	164	100	67	180	1.82	20				55	1.0
1400	22	Female	168	278	60	145	88	88	150	1.91	39	4.1	2.2		65	0.8
1761	26	Female	170	254	67	137	92	69	180	1.86	21	4.0	3.2	0.9	50	2.8
1664	27	Female	165	260	63	137	87	79	165	1.90	29				58	1.9
1718	33	Male	189	278	68	157	107	65	173	1.77	22	4.9	3.7	0.7	55	0.7
1727	40	Male	198	300	66	145	95	90	172	2.07	30	4.9	3.0	1.3	60	3.7
1801	42	Male	208	377	55	206	114	69	175	1.83	23	4.4	2.6	1.2	62	0.9
1549	49	Male	213	383	56	168	93	104	180	2.28	32				55	2.1
1750	51	Female	150	227	66	148	98	51	167	1.54	18	3.8	3.0	0.9	60	1.0
1600	51	Female	134	213	63	122	76	68	163	1.75	26	4.2	2.8	0.7	50	0.8
1739	52	Male	213	352	61	168	102	90	175	2.09	29	4.1	2.6	0.8	65	0.7
1666	54	Male	159	262	61	152	92	62	173	1.73	21			1.2	65	0.8
1622	56	Male	156	259	60	154	93	66	155	1.69	28	4.2	2.4	0.9	65	1.3
1690	63	Female	137	247	55	153	85	56	167	1.61	20	3.6	2.6	0.8	60	0.9
1716	64	Female	158	269	59	139	82	81	166	1.93	29	3.9	2.3	0.9	70	0.9
1490	71	Female	104	206	50	142	72	48	158	1.45	19					0.5
1732	72	Female	156	271	58	159	91	67	157	1.71	27	4.0	2.9	0.8	65	0.6
1485	78	Male	234	448	52	212	111	92	175	2.12	30					3.4
1488	81	Male	218	402	54	210	114	78	170	1.91	27					1.6

Patient ID #	Category	Age (year)	Gender
20998065	CT	81.6	Female
10315029	CT	87.7	Male
20875195	CT	74.1	Female
21000504	CT	85.9	Female
21001933	CT	80.8	Female
20240514	CT	89.7	Female
11331231	CT	81.1	Male
11615242	CT	71.6	Male
10589255	CT	74.3	Male
15196262	CT	81.1	Male
